# Hedonic and Utilitarian Motivations of Home Motion-Sensing Game Play Behavior in China: An Empirical Study

**DOI:** 10.3390/ijerph17238794

**Published:** 2020-11-26

**Authors:** Yuqi Liu, Yao Song, Ryoichi Tamura

**Affiliations:** 1 School of Design, Kyushu University, Fukuoka 8158540, Japan; tamura@design.kyushu-u.ac.jp; 2 School of Design, The Hong Kong Polytechnic University, Hong Kong 999077, China

**Keywords:** motion-sensing game, wellness and health, Chinese market, motivation, empirical study

## Abstract

As an important branch of video games and the integration of emerging motion-sensing technology, home motion-sensing games cannot only bring hedonic entertainment but also promote utilitarian benefits including exercise and social interaction for people to improve their physical and psychological health. As one of the most populous countries in the world, China has the largest number of households in the world but quite a low home game penetration rate due to the 13 year game industry winter for international enterprises. Whether Chinese customers have the intention of using motion-sensing games to improve their health status in the home environment will directly determine the commercial potential of the relevant industry in the Chinese market. In order to understand the motives of users and explore the market possibility and prospects of the game industry, this study adopts empirical research and structural equation modeling to construct a motivation model of Chinese consumers toward motion-sensing gameplay behavior in the household environment. We distributed 515 questionnaires to conduct a survey; 427 valid responses have been received, and 203 data, which meet the inclusion criteria of the required game experience, have been analyzed by SPSS25.0 and AMOS25.0. A structural equation model for the gameplay motivation has been constructed. The result shows that the three functional motivators, exercise (Path efficient = 0.40, *p* < 0.01), entertainment (Path efficient = 0.27, *p* < 0.01), and social interaction (Path efficient = 0.36, *p* < 0.01) of home motion-sensing games have a significantly positive impact on the user’s intention to play. Furthermore, the diversity and the time-and-place flexibility variables exert an important positive influence on the users’ gameplay behavior through their effects on the three main functional motive variables. To sum up, (1) exercise, (2) entertainment, and (3) social interaction are the main functional motivations of the Chinese consumers’ gameplay behaviors; (4) diversity and (5) time-and-place flexibility are the two main attribute motivators. The acceptance of Chinese users for home motion-sensing games remains positive and high. The motion-sensing game industry has broad market prospects in China through its potential in promoting consumer’s wellness and health in the home environment.

## 1. Introduction

Home is one of the places where people spend the longest time in life. How to maintain a happy and healthy home life has been drawing high concerns through the ages and will receive continuous attention in the future [1]. Due to the worldwide spread of COVID-19 coronavirus [2], hundreds and thousands of occupational and leisure activities have been transferred to the indoor and household environment because of the forcing requirements of social distancing and self-isolation. People’s activity space has been limited, and the risks of developing extra physical and mental health problems such as overweight, anxiety, depression symptom, denial, anger, and fear have been raised rapidly [3]. Furthermore, in the post-epidemic era, the changes that the pandemic brought in people’s way of living and working will exert a persistent and profound impact on our daily life. A regular exercise routine and pleasure lifestyle is the key to maintaining physical and psychological health during a forced tough period due to the COVID-19 pandemic emergency as well as the whole lifespan [4,5]. Motion-sensing gameplay is an emerging branch of video games, it breaks through the conventional gamepad and button input method of electronic games by using the full-body control to realize the interaction between player and game environment. Because of this character, motion-sensing games not only have similar entertainment and social characteristics as conventional video games but also can be used for fitness and exercise, and even medical rehabilitation, due to their body interaction models [6], which indicate that it can be compatible with the exercise [7], social interaction [8], and entertainment benefits and has a wide range of application in the health industry [9]. The benefit groups can range from children and adolescents [10] to elderly people [11], not to mention adults. Like a typical way of living a healthy lifestyle, especially in the context of the global COVID-19 pandemic, its values in maintaining physical and psychological health in the indoor environment become increasingly prominent and attractive.

Motion-sensing games have two main application scenarios: One is in the public commercial environment such as a large game console hall, a museum display to conduct education interaction, a workplace to relax staffs’ pressure, and a nursing home to conduct medical rehabilitation, etc. Another is in the home environment, and this scenario has broad market prospects due to the huge population and family bases in China. The most popular motion-sensing game company and devices worldwide until now are Nintendo Wii (released in 2006), Sony PlayStation Move (released in 2010), Microsoft Xbox, and Kinect (released in 2010) [12]. However, it is worth mentioning that during the heyday of the world’s motion-sensing games industry, the performance of international game enterprises in the Chinese market was almost blank. This is because the State Council of the People’s Republic of China issued the “Opinions on launching special governance of video game business premises” in 2000, which officially closed the door of the domestic game console industry and made the local gaming market enter a 13 year winter. In 2014, the State Council of the People’s Republic of China announced a notice, which clearly stipulated that foreign-funded enterprises were allowed to engage in the production and sales of game equipment in the Chinese domestic market with the condition that the relevant products had passed the content review and received permission from the cultural authority. This means that the 13 year ban on game device sales was officially lifted in China. The absence of the motion-sensing games golden age has directly led to the extremely low market penetration rate of this game industry in China up until now. However, with the increase in the material, cultural, and spiritual needs of the Chinese people, the related game industry will obtain much better market performance and huge business opportunities in China compared to the former period. Statistic data from Jingdong, a mainstream electronic products e-commerce platform in China, show that during the Spring Festival in 2020, the Chinese consumer’s demand for gaming devices was 254% of the same period last year. Among them, Sony PlayStation 4 sales increased by 133%, Microsoft Xbox sales increased by 188%, and the Nintendo Switch accounted for nearly 50% of the whole gaming devices sales, ranking first in popularity. The sales of the “Ring fit adventure” fitness game, which was released in 2019 from Nintendo, has also soared: its sales have increased by 363% during the COVID-19 pandemic period in China. For “Ring fit adventure”, with the elastic circular controller Ring-Con and the leg strap Joy-Con, the player’s steps and knee bends can be captured during the interactive gaming process. These two devices cooperate together to monitor the activities of different body parts of the player and realize the somatosensory function. People choose “Ring fit adventure” for the motivation of multi-dimensional needs, such as generating positive emotion, getting themselves moving for fitness, enhancing interaction with family members, seeking freshness, and so on. Furthermore, under the influence of the COVID-19 pandemic, exercise, entertainment, and social interaction have become unprecedentedly important needs for people to keep healthy, while staying home for a long time, and the “Ring fit adventure” can cultivate and develop the players exercise interests and fitness habits at home in a pleasant way for them to lead a healthy lifestyle. It also appeared on China Central Television and official comment announces that green and healthy video games have become an emerging demand for the general Chinese public.

In summary, motion-sensing games have the dual characteristics of hedonic and utilitarian applications. This study takes the motion-sensing games as the research object, mainly focusing on their application in the home environment, and takes the world’s largest market, the Chinese customer group, as the targeted audience to conduct an empirical study about the motives of Chinese gameplay behavior toward home motion-sensing games, so that their purchasing and playing motivation mechanism can be seen, and the market potential and prospects of motion-sensing games industry could be revealed. Through a literature review on game motivation combining with the cognition of the functional value and the game attributes, we refined six variables, namely entertainment, exercise, social interaction, diversity, time-and-place flexibility, intention to play, and proposed nine hypotheses based on the logical interaction effect analysis of the six variables. After the accomplishment of the questionnaire, which mainly consists of the demographic information and the variable measurements, we distributed 515 copies to conduct the questionnaire survey; 427 valid responses have been received and 203 relevant data, which meet the inclusion criteria of the required game experience, have been analyzed by SPSS 25.0 (IBM, Armonk, NY, USA) and AMOS 25.0 (IBM, Armonk, NY, USA). Path analysis and structural equation modeling were applied, the motivation model of Chinese users has been formulated and verified. The result shows that the acceptance of Chinese customers for home motion-sensing games stays positive and high, (1) exercise, (2) entertainment, and (3) social interaction are the main functional motives; (4) time-and-place flexibility and (5) diversity are the two main attribute motivators of the Chinese consumers’ purchasing and playing behaviors. The game industry has broad market prospects in China through its potential in promoting user’s wellness and health in the home environment. This study complements the current gap in Chinese user motion-sensing games motivation research. The result reveals the market prospects and driven mechanism behind the Chinese consumers playing and purchasing behaviors at an empirical level and provides valuable references for the product development and enterprise expansion strategy of the relative industry in China.

## 2. Literature Review

### 2.1. The Development Status of Motion-Sensing Games

Motion-sensing gameplay is a new type of video games. It breaks through the traditional operation mode of the gamepad button and equivalent gear and realizes the interaction between the players and the game system and the environment through physical body movements [13]. In 2006, the Japanese game company, Nintendo, released a new generation of game console Wii, which introduced motion-sensing technology to video game consoles for the first time and provides affordable motion-sensing devices for the mass market, opening the way for the motion-sensing games industry [14]. It allows players to use their full body as the controller of game input and enjoy the unique somatosensory interaction experience with the game system. After 2011, motion-sensing games can simulate three-dimensional scenes. At present, there are three most popular and low-cost commercially available motion-sensing game devices in the world: Nintendo’s Wii (released in 2006), Microsoft’s Kinect (released in 2010), and Sony PlayStation Move (released in 2010) [15].

Nintendo has been trying to launch motion-sensing games and researching the business of home fitness through games in every era. In 2006, Nintendo began to create the “Wii” series of motion-sensing games by attracting players with the mode of “playing outdoors at home”. It is an epoch-making product in the game industry and changes the game input from finger movement to full-body movements, making game fitness possible [14]. Moreover, the multiplayer modes of the game system also help players realize social interaction with their friends and family members through gaming [16]. Among them, the “Wii Fit” released in 2007 was welcomed by women consumers and had a total sales volume of over 20 million. Its Wii Ballance Board can support yoga exercises at home. Some studies even use this board to develop rehabilitation games for people with neurological injury [17]. After that, Nintendo continued to make a breakthrough in the motion-sensing games field, successively releasing “Fitness Boxing”, “Just Dance”, “ARMS”, “Super Beat Sports”, “Mario Tennis Aces”, “Sports Party”, “Zumba Burn It Up”, and other well-known and popular somatosensory games. These games are integrated with various sports activities, including racing, boxing, dance, music, yoga, tennis, and golf [18] and have multi-functions such as entertainment and leisure, family interaction, sports, and fitness. “Ring Fit Adventure”, which was released in 2019, is the latest fitness game on the Nintendo Switch platform. The game was sold with fitness ring accessories together. In 2020, because of the global COVID-19 epidemic, indoor fitness and entertainment demand is growing rapidly due to the continuous home living mode, so the price of “Ring Fit Adventure” has soared. The commercial success even resulted in a shortage of supply due to the huge market demand according to the official announcement of Nintendo.

Kinect is a motion-sensing input device developed by Microsoft for the Xbox 360 video game console and Windows PCs [19]. Being different from Nintendo Wii, players do not need any gamepad or handheld device to operate it [20]. As long as you stand in front of the camera, Kinect can recognize your movements and accurately project them on the game screen [21]. Through the cooperation of the built-in infrared camera, various sensors, and microphones embedded in the machine, players are truly freed from the controller, and even the Xbox can be controlled by voice and gestures [22]. Microsoft motion-sensing device, Kinect, has ever been certified by Guinness Records as the fastest-selling consumer device in the world. It sold 8 million units in 60 days after it was released in the market. However, due to the cruelty of host wars in the last century, in order to seize the market, both Microsoft and Sony sold machines at a loss and used the licensing fees of game software to get money back. Kinect, even equipped with a plenty of powerful and black technology, only sold for USD 149. Thus, no matter how popular it was in the game market, statistical data indicate that Kinect eventually sold more than 30 million units, but the hardware was not profitable at all. What makes matters worse, there were no core applications on the Kinect system to support such tremendous amount of sales, not to mention “Fruit Ninja”, “Dance Central”, “Kinect Sports”, or “Your Shape Fitness Evolved”; they all had a certain appeal power, but in the end, they were still leisure games and could not make players maintain freshness and enthusiasm for a long time. In the practical application of Kinect, some factors also restricted its development. For example, standing distance has an important influencing effect on the interaction efficiency [23]. For a Kinect-based game, the player needs to make at least a 2 × 2 m space in the living room, without any obstructions in the middle, so that Kinect can accurately identify the player’s body. For American families in a sparsely populated area, most of them have to move their furniture to ensure enough space before playing the Kinect game, not to mention the East Asian country’s households. However, Microsoft arbitrarily believed that Kinect is the future of the game, and even when the next-generation console Xbox One was released, Microsoft forcibly bundled Kinect 2.0 with it for sale, and the price surpassed USD 100, which was more expensive than the Sony PlayStation 4. In the end, because the hardware was not profitable enough, there was not enough lethal game software, developers remained unenthusiastic, core players were not interested, and a large number of non-core players left, signaling the wrong estimation of the future situation of the game: even though Kinect had been a big commercial success, it quickly fell from the altar. Microsoft had to announce the unbundling of Kinect and Xbox One, stopped production and officially declared the end of its somatosensory era. Although we have not seen Kinect appear in the game field independently for a long time, its infrared recognition technology still has very broad application prospects in the fields of game industry, civilian use, scientific research, including virtual reality gaming, healthcare, education, digital arts, retail services, robotics control and interaction, sign language recognition, safety training, etc. [21], such as the iPhone X camera application. We have the reason to believe that when Kinect becomes more convenient, accurate, and cheaper, it will return to the players’ world again.

PlayStation Move is a new generation of somatosensory equipment from Sony, a somatosensory motion controller introduced during the PlayStation 3 period. Its principle is similar to the Nintendo Wii and Microsoft Kinect, having inserted motion-sensors, LED lights, and a motion-sensing camera. The camera is used to track the position of the LED light for somatosensory operation. PlayStation Move cannot only recognize up and down, left and right movements of players, but also the changes in the angle of the wrist [24]. The dynamic controller can also sense the depth of the space, making the player feel as if they integrate with the game environment and experience a realistic and relaxing game experience. There are not many motion-sensing games on PlayStation Move. Typical representatives are “Just Dance”, “Fruit Ninja”, “NBA2K17”, etc. It can be compatible with some Kinect games, but in terms of motion-sensing games, Nintendo Wii and Microsoft Kinect are far more predominant than Play Station Move, no matter the aspects of the richness of game types or game operation experience [25]. Based on PlayStation Move, Sony has been developing PlayStation VR. Currently, motion-sensing games in the VR field are still immature, but we can recognize the trend of integrating emerging smart technology, such as augmented reality and virtual reality, in the motion-sensing game field according to the development direction of Nintendo and Sony.

In addition to the mainstream game industry, motion-sensing games have also attracted the attention of other commercial industries, such as the sports goods industry, fitness industry, television industry, medical industry, aging industry, etc. Nike has been innovating in the field of sports digitalization, for example, applying software and hardware services such as “Nike+ Running”, “Nike+ Fuel Band”, and “Nike+ Watch” to motivate athletes and players. “Nike Kinect Training” is a part of the Nike+ series [26]. In this game, players will conduct realistic system training under the leadership of Nike’s ace coach. The coach observes the player’s actions during the course, tells the player whether the action is standard via real-time feedback, evaluates the player’s physical fitness and exercise intensity based on the exercise results, finds the shortboards for improvement, and sets up a personalized fitness plan. What is more, “smart fitness equipment” refers to the integration of motion-sensing games and fitness equipment. It is a diversified game interactive content composed of multiple types of fitness equipment. Taking spinning as an example, its principal is to collect the speed frequency and drag coefficient in the spinning bike, transmit it with the wireless signal, receive the signal by the receiving device on the computer side, and convert the signal into an electronic signal, which is judged and calculated by the software to realize the function of interactive competition. This type of equipment also belongs to the category of motion-sensing games and can promote the user’s health both physically and psychologically [27]. It is worth mentioning that, following the success and experience of international motion-sensing game developers, the local motion-sensing gaming products in China have also been gradually emerging, such as Asus Wavi Xtion (released in 2011), Eedoo green 3D motion-sensing game console (released in 2012) and Shenzhen Taishan online Idong motion-sensing exercise machine (released in 2012), etc.

### 2.2. The Application of Motion-Sensing Game in Health

Motion-sensing games have characteristics of authenticity, sportiness, and interactivity. These characteristics make them extremely useful in promoting users’ psychological and physical health and share effectiveness and potential in a wide range of healthcare applications [28]. From the insight of existing successful motion-sensing games products, the trend of integrating fitness and sports games with hardware was quite obvious and received a far better market performance than those leisure game types [29]. Home motion-sensing games introduce the game products to home and indoor scenarios, providing infinite possibilities for shaping a healthy family lifestyle. Through them, users can simultaneously acquire the benefits of game playing, sports simulation, physical exercise, disease prevention, medical rehabilitation, etc. [22,30]. Moreover, some studies show that game therapy has better effects on autonomy, presence, and functional reach test comparing with conventional therapy [31]. In summary of the existing research, motion-sensing games could mainly be conducive to enhancing people’s abilities in the following four aspects, namely motion control ability, cognitive ability, emotion and willpower, and social ability. For each kind of ability problem, they can help with disease prevention, treatment, and carrying out some certain rehabilitation. Among them, motion control ability and cognitive ability belong to physical health application, emotion and willpower and social ability belong to mental health application. People who apply these games to conduct diseases prevention, treatment, and rehabilitation training can range from children to the elderly.

- *Motion control ability*. The motion control ability mainly refers to the function of the motor system and the nervous system of individuals. The related body parts and organs include eyes, hands, feet, spine, waist, breech, etc. Motion-sensing games can benefit the users in perceived motor competence and motor skill competence [32], hand and foot coordination [33], reaction time [34], balance training [35,36], fall prevention [37], chronic stroke [38,39], muscle function [40], Parkinson’s disease [9] etc.
- *Cognitive ability*. The cognitive ability is mainly reflected by the individual’s perception [41], attention [42], understanding [43], logical thinking [41], etc. Motion-sensing games could be utilized to not only enhance cognitive functioning (e.g., cognitive flexibility, working memory) [44], but also deal with a wide range of cognitive diseases including the chronic spinal cord [45], traumatic brain injury [45], chronic poststroke hemiparesis [46], cerebral palsy [47,48], mental retardation [49], autism [50,51], attention-deficit and hyperactivity disorder [52], mild cognitive impairment [53], and Alzheimer’s disease [54], etc.
- *Emotion and willpower*. Even though physical health is of great importance and always gains commercial priority in the health industry, psychological health also plays a critical role in maintaining human body health, which should not be ignored. Some studies reveal that people’s psychological health level could directly influence physical health status [55]. Thus, keeping positive emotions, getting the negative emotions under control, and relieving the pressure in a timely manner, etc., are important for maintaining a healthy lifestyle [56]. Motion-sensing games, with the video game essence, have the inherent entertainment and amusement nature to help people gain positive emotion.
- *Social ability*. Humans are a kind of social animal [57]. Social ability mainly contains personal empathy, communication, and interaction skills with others. It is a significant capability for an individual to build connectivity with the external world. Motion-sensing games could benefit people by strengthening relationships and building connections with strangers, friends, and family members through its online and offline game environment, especially in the home scenario, its low play barrier, multi-player mode, and strong interactivity could bring children, parents, even grandparents together to promote the intergenerational interaction [58,59].

### 2.3. Research on Game Motivation Model

To study and analyze the motivation model of Chinese users toward home motion-sensing games, the authors have reviewed relevant literature from previous studies. There are quite a number of scholars who try to develop scales to measure the motivation of game playing behaviors. Lafreniere has developed a multidimensional scale of gaming motivation in line with self-determination theory called the gaming motivation scale (GAMS); six constructs have been confirmed, they are intrinsic motivation, integrated, identified, introjected, and external regulation, as well as amotivation [60]. Adam has jumped out the limitation of game motivation research focusing on specific game genres or player cultures as well as the lack of behavioral validation, offered a new scale and evaluated its validity in a cross-genre, cross-cultural, and behaviorally validated perspectives; six types of player motivation have been found, they are socializer, completionist, competitor, escapist, story-driven, and smarty-pants [61]. Ju-hui Chang has analyzed the online game, Chinese players, from materialism and motivation to attitude; five motivation items have been used, including self-confidence and achievement, escape and virtual identification, sociality, entertainment, and reward [62]. De Grove has developed an instrument for measuring individual motives of digital game players from a social cognitive theory perspective, and the scale includes eight constructs, namely habit, moral self-reaction, agency, narrative, escapism, pastime, performance, and social [63]. He also has used the digital games motivation sale (DGMS) to test game usability between different cultures and countries and presented a psychometrically sound measurement instrument that can be used in cross-cultural settings [64]. However, the majority of game motives research up until now has been geared towards conventional games from a macro perspective, such as video games, online games, and digital games, and a few of them are geared towards emerging technology games. Agnes Zsila has expanded motives of the online gaming questionnaire, added three new factors—outdoor activity, nostalgia, and boredom—to conduct a separate scale study on “Pokémon Go”, an augmented reality game, which is supposed to be the first empirical contribution to the assessment and understanding concerning the motives of augmented reality gameplay [65]. Hsin-hui Lin has conducted empirical research on motivation for physical game systems use behavior from both hedonic and utilitarian aspects. The research constructs include performed exercise utility, perceived enjoyment, perceived motion-sensing, ease of use, interactivity, design aesthetics, challenge, and behavioral intention [66]. The direction of its thoughts and the selection of variables are profound and convincing, but it ignores the importance of social interaction in games, and the research is aimed at regular physical games; it has not analyzed the specific market of motion-sensing games, and there is a lack of the motivation and market potential analysis in the household scenario. To sum up, there are a quite number of researches try to measure the motivations underlying gameplay behaviors from different theoretical perspectives, but most of them are concentrated on conventional game types. At present, there are few research targeted at motion-sensing games. Moreover, as one of the largest markets in the world, Chinese consumers’ motivation and market acceptance toward gaming has an important impact on the development and revenue of the relevant game industry. With regard to the game form embedded emerging technologies, such as motion-sensing technology, virtual reality, augmented reality, etc., the majority of them are applied in public commercial scenario, but the home application scenario, which is a mass market with the largest consumption crowd basis, should be given great concerns both currently and in the coming future. Thus, the goal of present research is to conduct an empirical study to obtain deeper understanding about the motivation of motion-sensing gameplay behavior and construct a motivation model to reveal the underlying logic of Chinese customers gaming intention in a household scenario. The result can provide important and valuable reference for the product development and market expansion of the relevant game industry in China.

## 3. Hypotheses Development and Research Framework

Following the theoretical review and the analysis of application and development status of motion-sensing games, we selected six motivation variables as our research object to construct the motivation model of Chinese customers, they are exercise (EXE), entertainment (ENT), social interaction (SI), time-and-space flexibility (TSF), diversity (DIV), and intention to play (IPL). Due to the deduction based on existing facts and research, nine hypotheses have been proposed, and the research framework has been formulated.

### 3.1. Exercise

The exercise (EXE) motivation refers to the users’ intention of gaining the benefit of fitness and health through gameplay behavior. To be more specific, within the context of motion-sensing games [66], exercise motivation refers to the physical activity for sustaining or improving mental and physical health. There is plenty of motion-sensing games that can simulate dance [67], music [68], yoga [69], boxing [70,71], tennis [72], golf [18], and many other popular sports. They integrate the functions of sportiness and fitness with the game products, so that people can reach the purpose of doing exercise without leaving home and keep fit in a pleasant way. For example, “Ring fit Adventure”, launched from Nintendo, is a game that combines fitness and amusement effectiveness. Its low barrier to play and friendly interaction format allows players to exercise in a relaxing and happy atmosphere and develop a good habit of fitness to live a healthy lifestyle in indoor environment. Due to the former insight, we formulate the hypothesis below.

**Hypothesis 1** **(H1).** *Exercise can positively affect the intention to play.*

### 3.2. Entertainment

The entertainment (ENT) motivation is mainly about the users’ intention to apply motion-sensing games as a way of having enjoyment and amusement to gain positive emotion such as being relaxing, happy, excited, satisfied, etc. As an important branch of video games, it has an inherent entertainment nature due to its game essence. Moreover, differing from the conventional video games, motion-sensing games use Wii, Kinect, Eyetoy, and other somatosensory recognition devices to capture the user’s body motion, which can provide real-time feedback of any detailed movements of the user to the somatosensory game running equipment. They allow users to use different parts of their body directly or combining with simple sensor devices to realize real-time interaction with the game environment and the control of the game characters, which integrates the feeling of novelty, fun, appreciation, high-technology, etc., which have a strong sense of presence and realism to bring players an interesting, exciting, and real gaming experience [73]. Moreover, motion-sensing games are also showing the development trend of combining AR [74], VR [75,76], MR [77], and other advanced technology, as well as developing more accurate and efficient motion-sensing equipment and wearables [78], which will bring players far more immersive experience and all-round perception of entertainment in the future. Thus, we propose the following hypothesis.

**Hypothesis 2** **(H2).** *Entertainment can positively affect the intention to play.*

### 3.3. Social Interaction

Social interaction (SI) motivation is defined as the users’ intention to exchange experiences and building real or virtual connectivity with other individuals through the way of gaming. Motion-sensing games have strong interactivity capability, which can be revealed from the following two aspects. On the one hand, the players could have a strong interaction with other people via the virtual game characters as well as the game environment through wireless signal transmission with accuracy and validity rate of physical action signals [79]. On the other hand, motion-sensing games are products that embody motion-sensing technology, through which people can directly use body movements to interact with surrounding devices and the environment. It could provide a multi-player mode allowing players to play with friends and family members, which is useful to strengthen their relationship bonds and promote interaction and emotional communication [16]. Due to the low operation barriers of motion-sensing games, users from all different ages can find their favorite game types, which means it can also promote intergenerational interaction (e.g., children and their parents or grandparents). Based on the analysis above, we put forward the hypothesis below.

**Hypothesis 3** **(H3).** *Social interaction can positively affect the intention to play.*

### 3.4. Time-And-Space Flexibility

The time-and-space flexibility (TSF) motivation refers to the attribute of home motion-sensing games allowing the users to play the game in an indoor and private environment at any time they want, which helps the customers get rid of the limitation of business hours to go to the certain public or commercial venues (e.g., gym, stadium, game center, etc.). Home scenarios could provide great freedom and flexibility in time and space for game players, especially those who have a busy schedule and feel hard to follow the business hours of typical public commercial places. This attribute provides huge convenience and indicates typical advantage to promote exercise, entertainment, and social interaction activities for users at home. Taking these facts into consideration, the authors formulate the following hypothesizes.

**Hypothesis 4** **(H4).** *Time-and-space flexibility can positively affect exercise.*

**Hypothesis 5** **(H5).** *Time-and-space flexibility can positively affect entertainment.*

**Hypothesis 6** **(H6).** *Time-and-space flexibility can positively affect social interaction.*

### 3.5. Diversity

The diversity (DIV) motivation is considered as the richness of motion-sensing game types, play mode, content, and experience. Customers are disloyal and always try to pursue freshness [80]. It is hard for them to keep consistent enthusiasm for a certain product or service that stays unchangeable for a long time. The failure of Microsoft Kinect and the success of Nintendo Wii in the game field, which we mentioned in the former content, also prove this insight. In other words, the diversity of motion-sensing games’ settings and experiences is one of the key elements to the commercial success of gaming products. Richness could tremendously benefit and provide diverse experiences for the users’ exercise, entertainment, and social interaction activities. The game, “Ring fit Adventure”, which has achieved huge commercial success and received a wide welcome, adopts an adventurous form, provides more than forty kinds of sports with various exercise challenges for different body parts, and is equipped with a relaxing mode. The reason why it received high praise in the game market is not only because of its fitness market positioning but also its richness of game content and experience. Due to the analysis above, we suppose that the diversity variable should have a positive influence on exercise, entertainment, and social interaction motivation.

**Hypothesis 7** **(H7).** *Diversity can positively affect exercise.*

**Hypothesis 8** **(H8).** *Diversity can positively affect entertainment.*

**Hypothesis 9** **(H9).** *Diversity can positively affect social interaction.*

**Figure 1:**
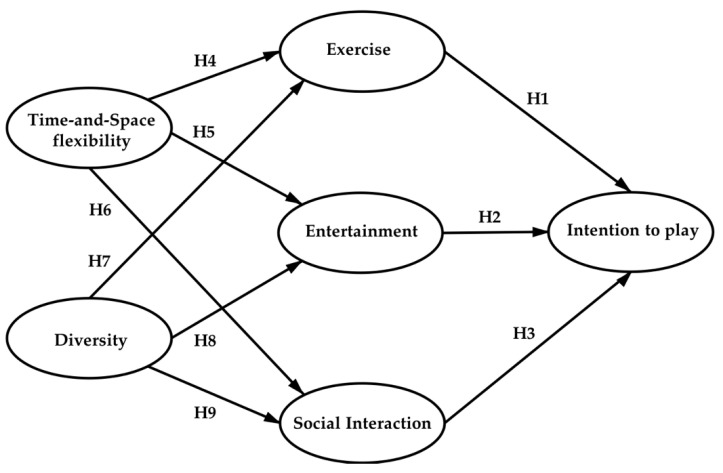
The conceptual framework of hypotheses.

According to the description and analysis above, the nine hypotheses and research framework have been developed, as shown in Figure 1. We argue that exercise, entertainment, and social interaction are the three main functional motivators for customers’ intention to play, while diversity and time-and-place flexibility are the main attribute motivators; they play positively significant roles in promoting exercise, entertainment, and social interaction functions of home motion-sensing games, which contributes to the intention to play at the end.

## 4. Methodology

To validate the conceptual framework shown in Figure 1, in the following part, an empirical study was implemented to examine the relationship between different motivation variables in the model.

### 4.1. Measurement Development

In terms of construct measurement, Table 1 summarizes the measurement items that were adopted to test Hypotheses 1–9. To be more specific, six measurements related to this model were retrieved from prior studies and were formatted on a five-point Likert scale for this research.

### 4.2. Survey Procedure and Data Collection

The survey procedure contained two phases to ensure the questionnaire was appropriately designed to address the research questions: a pilot study and the main study.

First of all, a sample of 30 participants at a major university in central China was enrolled for the pilot study. By revising the questionnaire, we removed the redundant questions and ensured the survey as understandable and concise as possible. Accordingly, the revised survey contained 23 items that contained not only measurement items mentioned in Table 1 but also the demographic information and general attitude questions toward home motion-sensing games, as shown in the Appendix A.

Next, we distributed the revised survey via 12 counselors from the same university in the summer of 2020. A total number of 515 questionnaires were spread to the teachers, students, and their family members from various regions. To specify, participants with related game experience were set as the inclusion criteria. Of the 427 responses received, 203 unique surveys that met the inclusion criteria were valid for the purpose of this study, leading to a 39.4% valid response rate, which were processed for further analysis.

### 4.3. Data Analysis Plans

As for data analysis, the research initially directed the descriptive analysis, reliability, and validity test. After confirming the satisfactory level of internal consistency, unidimensionality validity, convergent validity, and discriminant validity, we performed the SEM to examine the model fit and path analysis for hypothesis evaluations. To be more specific, SPSS 25.0 and AMOS 25.0 were used to conduct statistical analysis. SPSS was mainly responsible for summarizing the demographical information and the preliminary statistics, while AMOS was mainly used to conduct factor analysis and path analysis.

### 4.4. Demographic Information

As for the demographic information and attitudes towards the home motion-sensing game, 203 participants are enrolled in the current study. Detailed information could be found in Table 2.

## 5. Results and Findings

### 5.1. Descriptive Analysis, Reliability, and Validity

The reliability of a scale indicates the degree that items reflect similar observations under similar scenarios with similar participants [87]. Cronbach’s alphas evaluate the internal consistency of the survey with a threshold of 0.7, as shown in Table 3. Results showed all the factors achieved a satisfactory level of reliability [88]. Similarly, construct validity shows the degree of association between a claimed factor and its operationalization, which included three parts: unidimensionality validity, convergent validity, and discriminant validity.

Unidimensionality validity aims to examine whether a measurement has only one single dimension via confirmative factor analysis (CFA) [89]. Results showed all the 18 items of six factors evaluated as they were planned. Table 3 summarizes the reliability and unidimensionality of all six factors.

Convergent validity provides the degree that six factors are associated with each other theoretically and practically. According to previous work [90], the threshold of standardized factor loadings and averaged variances expected (AVE) are both beyond the threshold (0.5 or above), and the composite reliability (C.R.) should be above the threshold of “2”. According to the result, it indicated the C.R., standardized factor loading, and AVE are above the threshold, suggesting the current data satisfied the convergent validity. Besides, discriminant validity shows the scenario that factors that should not be related are unrelated in reality. This validity could be measured by correlation coefficients, maximum shared variance (MSV), and average shared variance (ASV). Table 4 indicated the MSV and ASV are also within the threshold (threshold: MSV < AVE and ASV < AVE), indicating that six factors achieved satisfactory discriminant validity.

### 5.2. Model Fit

As for model fit, the goodness-of-fit was measured by the following index [91]: standardized root mean square residual (SRMR), goodness-of-fit index (GFI), root mean square error of approximation (RMSEA), normed fit index (NFI), incremental fit index (IFI), Tucker–Lewis index (TLI), and comparative fit index (CFI) were generally within the respective thresholds, as shown in Table 5.

### 5.3. Hypothesis Testing and Path Analysis

**Figure 2:**
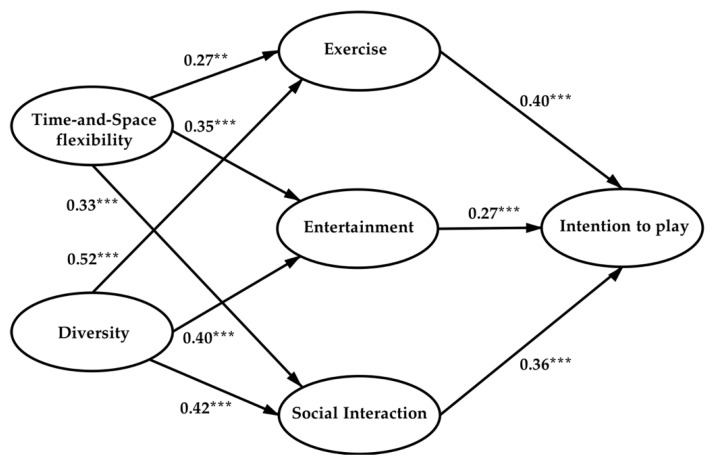
Path coefficients resulting from structural equation modeling (SEM). Note: ** *p* < 0.05; *** *p* < 0.01.

In order to examine the Hypothesis 1–9, a path analysis was conducted via structural equation model (SEM) to examine the relationship between various factors [92]. The reason to adopt SEM in this study is that it is a mature way to illustrate a holistic, and less blatantly causal, interpretation of research hypotheses in various studies [92]. Figure 2 and Table 6 show the results of path analysis and the appraisal of the Hypotheses 1–9.

## 6. Discussion and Implications

Differing from other countries in the world, the international game enterprises have experienced market winter in China from 2000 to 2013 due to the economic policy restriction from the State Council of the People’s Republic of China. It is since 2014 that the Chinese government began to open the domestic game market for overseas companies. Even though China has a tremendous population base and the largest market in the world, the motion-sensing games industry market penetration remains extremely low up until now. However, in recent years, there is a booming development trend of the motion-sensing games industry in China due to the loose policy environment and the public’s growing material and spiritual needs as well as fitness and health awareness, especially after the pandemic of COVID-19. This research takes the Chinese user group as the research object, conducts an empirical study to analyze their underlying motive mechanism of gameplay behavior, and explores the market potential of the motion-sensing games industry in China with its health and wellness application in the household scenario. Three functional motivators and two attribute motivators have been refined. The motivation model has been formulated. From the results, we can see that all the nine hypotheses proposed in this paper have been supported.

The intention to play variable is directly affected by the three main functional motivation variables—entertainment, social interaction, and exercise. The path coefficient of H1 is 0.40, H2 is 0.27, and H3 is 0.36, which shows that, for home motion-sensing games, users have similar entertainment motivation as conventional video games, and at the same time, their acceptance and demand expectations for the application of motion-sensing games in healthy exercise and family socialization have already been cultivated. Especially under the context of the global COVID-19 pandemic and the enforcement of social distancing and self-isolation, home exercise and indoor social interaction have become somehow a kind of just need. Motion-sensing games are the game form that has these unity functional benefits: the first reason is their essence as video games, thus they have the inherent entertainment advantage; the second reason is that they are different from traditional video games, getting rid of keyboards and handles and using the player’s body movements to input, interact, and control, which makes them have unique advantages in gaming exercise to maintain physical health; third, the virtual game environment and multiplayer game mode also provides the possibility to help the players strengthen relationships and promote interaction and communication with friends and families online and offline. In addition to the three functional motives, time-and-place flexibility is an important attribute motivation factor for intention to play. It directly affects the three functional variables of entertainment, social interaction, and exercise. In the analysis results, the path coefficient of H4 is 0.27, H5 is 0.35, and H6 is 0.33, which shows that the features of home motion-sensing games not limited by time-and-place have greatly facilitated users’ entertainment, social interaction, and exercise motivations in the household environment. Users do not need to be restricted by the business hours and space of commercial public places (e.g., amusement park, game center, social venue, gym, etc.) and can utilize their fragment time and home time to meet their leisure entertainment, healthy exercise, and family interaction needs through gaming, which greatly promoted their willingness to play. The result also indicates that developers and relative business enterprises should put their emphasis on enlarging the family scenario market of motion-sensing games, since China has the maximum number of households in the world but quite a low gaming penetration rate due to the 13 year absence of the global game industry market. Nowadays, the users’ consumption willingness and playing motivation remain strong. Last but not least, diversity has a significant positive effect on the three functional variables and indirectly affects the user’s motivation to play, the path coefficient of H7 is 0.52, H8 is 0.40, and H9 is 0.42, which shows that the diversity and richness of game types, mode, content, and experience, are important incentives for users to keeping freshness and persistent enthusiasm for gameplay behaviors. Contemporarily, the health applications and content of motion-sensing games on the mass market are still relatively single. Therefore, when developing related game products, developers should pay attention to the diversity and richness of game content and experience, optimize and update in time to earn users’ sticky, and stimulate their desire to play.

It is worth mentioning that home motion-sensing games products with health and fitness business positioning have a much more prosperous market and commercial possibility than regular leisure and entertainment games, which can be seen from the success of Nintendo Wii and the failure of Microsoft Kinect. However, in general, according to the review of development status, the application of motion-sensing games in the health field is still in its infancy stage and the optimization of the improvement of game content and user experience is necessary. Many games are designed with leisure aims, and not necessarily encourage players doing the exercise correctly; also, there are few types of commercial game types released toward a mass market for the healthcare purpose, and the evaluation of the health effect of gaming is still lacking systematic and feasible indicators, such as calorie consumption calculation and changes in physical indicators, to quantify and visualize the effect of gameplay. In addition, for certain diseases and training activities, the evaluation index needs professionally medical standards. What is more, the game operation and intensity should provide adjustable space for different severity levels and conditions of patients and individuals. Games might also be a good way to bridge the gap between fun and laborious training of disabled bodily functions, especially as a continuation of the training regimen after clinical sessions at home, which is strongly suggested. The current commercial games tested for rehabilitation in various literature with the possibility for continuation at home appear to lack a reliable way to track and motivate the progress of the patient, which the physical therapist would normally do in the clinical training session. To solve these difficulties and improve the application efficiency of motion-sensing games in the healthcare field, there is still a long way to go, but the market prospects are very broad, especially with the support and integration of emerging advanced technology.

To sum up, from the results of model verification, the motivation of Chinese users in utilizing home motion-sensing games as a typical form of home entertainment, social interaction, and indoor exercise has been strong, and the consumer awareness has initially been cultivated, which also means, at present and in the near future, the market of home motion-sensing games targeted on promoting physical and mental health in the household environment is prosperous and shows huge commercial potential in the Chinese market. Relative industries can take this commercial opportunity as a starting point to develop and improve the game experience, quantify and pinpoint gameplay effects and outcomes, and explore more possibilities for motion-sensing games in home scenario applications to achieve business success. Furthermore, in addition to the home environment, motion-sensing games can also expand their application of other indoor spaces in the form of health pursuit and fitness games, such as gyms, nursing homes, public fitness spaces for companies, enterprises, and organizations, etc. In terms of cross-field collaboration, motion-sensing games can also jump out of the setting of the game industry and integrate with other commercial enterprises such as the home appliance industry, sports industry, fitness industry, medical industry, and aging industry to expand its market potential and possibilities in health and wellness promotion.

## 7. Limitations and Future Studies

In this study, we mainly collected questionnaire answers through the same Chinese university, the valid responses are from teachers, students, and their family members, which to some extent stand for the high-educated group who use the Internet more often and comparatively have a higher acceptance of Internet-related smart technologies. At the same time, the volume of responses that meet the inclusion criteria of the required game experience is not particularly large, which also indicates that the penetration rate of motion-sensing games in the Chinese market is not high; the mass market is still in the initial stage of development, and relatively well-educated people are more receptive and motivated to use them. This group of people, which occupy a large proportion of the Chinese population, could be the targeted customers to open up the related gaming market. In the future, we will further improve our research from the following three aspects: first, we will expand the sample size returned by the questionnaire to enhance the universality and credibility of the conclusions; second, we will consider the influence of demographic variables on the intention to play, such as gender, age, occupation, marriage status, education level, etc., to study the difference of gameplay motives of specific groups, such as the elderly, student groups, office workers, etc., to explore the characteristics of different groups and their preferences for home motion-sensing games. Last but not least, this motivation model was mainly constructed based on the needs of users. For the attributes of home motion-sensing games, we only took the diversity and the time-and-place flexibility, two variables, into account, so there might be other potential variables that we have not been discovered. In the future, we will consider adding some new variables for more in-depth analysis and discussion.

## 8. Conclusions

Motion-sensing games are interactive video games that introduce motion-sensing technology and devices on the basis of conventional video games to improve the input method and realize game control through the whole-body movements of players. They have broad development prospects in terms of the application in physical and psychological healthcare. Home motion-sensing games aim at the mass market and provide greater freedom and flexibility for game players due to their application in a household scenario, which should help the business concerned and emphasize the relevant game industry. They can help users break through the constraints of time-and-place and meet their exercise, entertainment, and social interaction needs in an in-home environment. There are three reasons for their benefits. First, the game essence of a motion-sensing game determines its entertainment attributes. Second, low playing and operation barriers, as well as a wide range of game themes and materials, allow users from different ages to find their favorite game types with high playability, so there is no age limitation, and they are suitable for all age groups from children to the elderly. Simultaneously, the virtual game environment online and the multiplayer mode offline can help gameplayers promote social interaction with their friends and family members to strengthen emotional bonds. Third, the biggest difference between motion-sensing games and conventional video games is the full-body input way of game control, in addition to the benefits of improving emotional and psychological status, home motion-sensing games can also help users perform home exercises and be utilized as an effective way to deal with various health problems in their prevention, treatment, and rehabilitation stages as well as healthy knowledge education.

Although international game enterprises have gone through the 13 year market winter in China and China missed the golden age of global motion-sensing games development, which directly leads to its obvious lower market penetration compared to the developed countries, Chinese consumers’ willingness and enthusiasm for utilizing motion-sensing games for health improvement, including physical and mental aspects like entertainment, exercise, and social interaction nowadays, remain high. With urging pursuit of a healthy lifestyle and the expansion of social needs, users’ intention for motion-sensing games playing is not simply motivated by the entertainment demand, but also exercise and social interaction needs. These three types of needs have a significantly positive influence on the motivation of users’ playing behaviors. Furthermore, the time-and-place flexibility attribute of home motion-sensing games allows users to use their fragmented time to conduct gameplay in the home environment at any time they want, without the restriction of time and venue in the commercial space, which is one of the most obvious advantages for home motion-sensing games to seize the gaming market. Time-and-place flexibility could indirectly affect the intention to play variable through its effect on the three main functional motivation variables. In addition, the diversity of home motion-sensing games is also an important motive for users to play and maintain persistent enthusiasm. It has significant effects on the three main functional motivation variables, exercise, entertainment, social interaction, and indirectly affects the users’ intention to play. We can conclude that home motion-sensing games have broad market prospects in China, and exercise, entertainment, and social interaction are the three main functional motivations for Chinese users purchasing and playing behaviors. Time-and-place flexibility and diversity are the main attribute motivators that affect users’ functional requirements and the intention to play. With the development of advanced technology, the play and control methods of motion-sensing games will become more and more precise, rich, and diversified. It can also be integrated with emerging and cutting-edge virtual reality, augmented reality, mixed reality, extended reality technologies to bring users a far more authentic, immersive, and pleasurable game experience. Related industries should carry forward the time-and-place flexibility advantage of home motion-sensing games in the household and indoor environments; focus on the development of exercise, entertainment, and social interaction functions; explore the possibilities of motion-sensing games in the fields of health management and disease prevention, treatment, and rehabilitation; quantify the effect of gameplay and physical index evaluation standards; enhance their diversity and richness of game content, and optimize the game experience, to promote customers’ health and wellness, to maintain players loyalty and stickiness, and to achieve commercial success.

## Figures and Tables

**Table 1:** Constructs and measurement items.

Construct	Measure Item	Reference
Diversity (DIV)	DIV1: Motion-sensing games have rich game types and play modes.	[66,81,82,83]
	DIV2: Motion-sensing games have rich game contents.	
	DIV3: Motion-sensing games can bring me rich gaming experiences.	
Time-and-place flexibility (TPF)	TPF1: I can play home motion-sensing games without the time limit.	[83,84]
	TPF2: I can play home motion-sensing games without the place limit.	
	TPF3: I can begin and stop playing motion-sensing games at home anytime.	
Entertainment (ENT)	ENT1: I play motion-sensing games because it’s funny.	[66,82,83,84,85]
	ENT2: I play motion-sensing games because it’s cool.	
	ENT3: I play motion-sensing games because it’s exciting.	
Exercise (EXE)	EXE1: I play a motion-sensing game because it can help me to lose weight and sculpt my figure.	[66,81,86]
	EXE2: I play a motion-sensing game because it can help me to improve my physical health.	
	EXE3: I play a motion-sensing game because it can exercise different parts of my body via different control methods.	
Social interaction (SI)	SI1: When playing motion-sensing games with family members, it can help to promote the communication and enhance the emotional bonds.	[63,82,83]
	SI2: When playing motion-sensing games with friends, it can help to strengthen our relationship.	
	SI3: When playing motion-sensing games, I can know new friends.	
Intention to play (IPL)	IPL1: I am willing to play motion-sensing games.	[66,82,83,84]
	IPL2: I will try to play motion-sensing games.	
	IPL3: I will play motion-sensing games.	

**Table 2:** Demographic information and attitude for home motion-sensing game.

Attributes	Value	Frequency	Attributes	Value	Frequency
Gender	Male	85	Acceptance in Home Motion-Sensing Game	Low	6
	Female	118		Relatively low	14
Age	15–20	16		Medium	60
	21–30	122		Relatively high	99
	31–40	47		High	24
	41–	18	Future for Home Motion-Sensing Game	Pessimistic	4
Education	Some colleges	71		Relatively pessimistic	5
	Undergraduate	113		Neutral	19
	Postgraduate	19		Relatively optimistic	104
				Optimistic	71

**Table 3:** Reliability and unidimensionality.

Construct	Cronbach’s Alpha	Variable	Mean	Standard Deviation	Standardized Factor Loading	C.R. (t-Value)	SMC	AVE	Composite Reliability
Diversity (DIV)	0.824	DIV1 DIV2 DIV3	4.01 4.06 4.02	0.783 0.653 0.805	0.763 0.807 0.786	- 11.332 11.040	0.583 0.651 0.618	0.617	0.829
Time-and-place flexibility (TPF)	0.843	TPF1 TPF2 TPF3	4.18 4.08 3.98	0.638 0.786 0.660	0.784 0.800 0.842	- 11.753 12.396	0.614 0.640 0.719	0.655	0.850
Entertainment (ENT)	0.868	ENT1 ENT2 ENT3	4.07 4.01 3.97	0.805 0.802 0.783	0.774 0.925 0.805	- 13.510 12.063	0.598 0.855 0.649	0.701	0.875
Exercise (EXE)	0.823	EXE1 EXT2 EXT3	3.94 3.95 3.79	0.839 0.791 0.865	0.750 0.806 0.790	- 10.592 12.201	0.543 0.649 0.624	0.612	0.825
Social interaction (SI)	0.905	SI1 SI2 SI3	3.88 3.94 3.86	0.781 0.912 0.928	0.858 0.821 0.949	- 10.659 10.498	0.736 0.674 0.901	0.770	0.909
Intention to play (IPL)	0.848	IPL1 IPL2 IPL3	4.09 3.81 3.63	0.863 0.767 0.818	0.793 0.829 0.805	- 12.450 12.055	0.628 0.647 0.629	0.655	0.850

Note: C.R. (t-value) = composite reliability; SMC = square multiple correlations; AVE = averaged variances expected.

**Table 4:** Correlation matrix of the constructs.

	CR	AVE	MSV	ASV	IPL	DIV	TPF	SI	EXE	ENT
IPL	0.850	0.655	0.581	0.510	0.809					
DIV	0.829	0.617	0.610	0.513	0.762 ***	0.786				
TPF	0.850	0.655	0.610	0.488	0.749 ***	0.781 ***	0.809			
SI	0.909	0.770	0.472	0.374	0.687 ***	0.667 ***	0.647 ***	0.878		
EXE	0.825	0.612	0.507	0.410	0.712 ***	0.700 ***	0.646 ***	0.534 ***	0.782	
ENT	0.875	0.701	0.440	0.380	0.655 ***	0.663 ***	0.658 ***	0.497 ***	0.591 ***	0.837

Note: CR = composite reliability; AVE = averaged variances expected; MSV = maximum shared variance; ASV = average shared variance; IPL = intention to play; DIV = diversity; TPF = time-and-space flexibility; SI = social interaction; EXE = exercise; ENT = entertainment; *** *p* < 0.01.

**Table 5:** Goodness-of-fit test.

Category	Measure	Acceptable Values	Value
Absolute fit indices	Chi-square		195.561
	d.f.		125
	Chi-square/d.f.	1–5	1.564
	GFI	0.90 or above	0.906
	SRMR	0.08 or below	0.027
	RMSEA	0.05–0.08	0.053
Incremental fit indices	NFI	0.90 or above	0.922
	IFI	0.90 or above	0.970
	TLI	0.90 or above	0.963
	CFI	0.90 or above	0.970

Note: GFI = goodness-of-fit index; SRMR = standardized root mean square residual; RMSEA = root mean square error of approximation; NFI = normed fit index; IFI = incremental fit index; TLI = Tucker–Lewis index; CFI = comparative fit index.

**Table 6:** Hypothesis testing.

	Path Direction	Standardized Coefficient	Standard Error	C.R. (t-Value)	Result
H1	EXE → IPL	0.393 ***	0.089	4.819	Accepted
H2	ENT → IPL	0.267 ***	0.078	3.708	Accepted
H3	SI → IPL	0.355 ***	0.072	4.974	Accepted
H4	TPF → EXE	0.265 **	0.147	2.040	Accepted
H5	TPF → ENT	0.352 ***	0.141	2.822	Accepted
H6	TPF → SI	0.326 ***	0.148	2.672	Accepted
H7	DIV → EXE	0.519 ***	0.137	3.805	Accepted
H8	DIV → ENT	0.403 ***	0.127	3.183	Accepted
H9	DIV → SI	0.419 ***	0.133	3.371	Accepted

Note: TPF = time-and-space flexibility; DIV = diversity; EXE = exercise; ENT = entertainment; SI = social interaction; IPL = intention to play; ** *p* < 0.05; *** *p* < 0.01.

## Appendix A

The following is the main questionnaire we used in this study. It consists of two parts: the demographic part and the motivation measurement part. For each demographic item, the options have been included in the brackets below. For each measurement question, there are five answers that can be selected: ”Strongly disagree”, ”Disagree”, ”Neutral”, ”Agree”, and “Strongly Agree”.

### Appendix A.1. Demographics

Age (15–20, 21–30, 31–40, 41–), gender (male, female), education level (some college, undergraduate, postgraduate), general attitude (acceptance in home motion-sensing game on a 5-point Likert scale: low to high; future for home motion-sensing game on a 5-point Likert scale: pessimistic to optimistic).

### Appendix A.2. Measurement

#### Appendix A.2.1. Diversity (DIV)

- DIV1: Motion-sensing games have rich game types and play modes.
- DIV2: Motion-sensing games have rich game contents.
- DIV3: Motion-sensing games can bring me rich gaming experiences.

#### Appendix A.2.2. Time-And-Place Flexibility (TPF)

- TPF1: I can play home motion-sensing games without the time limit.
- TPF2: I can play home motion-sensing games without the place limit.
- TPF3: I can begin and stop playing motion-sensing games at home anytime.

#### Appendix A.2.3. Entertainment (ENT)

- ENT1: I play motion-sensing games because it’s funny.
- ENT2: I play motion-sensing games because it’s cool.
- ENT3: I play motion-sensing games because it’s exciting.

#### Appendix A.2.4. Exercise (EXE)

- EXE1: I play a motion-sensing game because it can help me to lose weight and sculpt my figure.
- EXE2: I play a motion-sensing game because it can help me to improve my physical health.
- EXE3: I play a motion-sensing game because it can exercise different parts of my body via different control methods.

#### Appendix A.2.5. Social Interaction (SI)

- SI1: When playing motion-sensing games with family members, it can help to promote the communication and enhance the emotional bonds.
- SI2: When playing motion-sensing games with friends, it can help to strengthen our relationship.
- SI3: When playing motion-sensing games, I can know new friends.

#### Appendix A.2.6. Intention to Play (IPL)

- IPL1: I am willing to play motion-sensing games.
- IPL2: I will try to play motion-sensing games.
- IPL3: I will play motion-sensing games.
